# Surgical decision-making in acute appendicitis

**DOI:** 10.1186/s12893-015-0053-x

**Published:** 2015-06-02

**Authors:** Eva Sandell, Maria Berg, Gabriel Sandblom, Joar Sundman, Ulf Fränneby, Lennart Boström, Åke Andrén-Sandberg

**Affiliations:** The department of Gastroenterology, Karolinska University Hospital, Stockholm, Sweden; The department of Gastrointestinal Surgery, Karolinska University Hospital, Stockholm, Sweden; Vårby General Practice, Stockholm, Sweden; Karolinska Institute, CLINTEC, Stockholm, Sweden; The department of Otorhinolaryngology, Karolinska University Hospital, Stockholm, Sweden; The department of Surgery, Södersjukhuset, Stockholm, Sweden

**Keywords:** Acute appendicitis, Signs and symptoms, Decision-making

## Abstract

**Background:**

Acute appendicitis is one of the most common acute abdominal conditions. Among other parameters, the decision to perform surgical exploration in suspected appendicitis involves diagnostic accuracy, patient age and co-morbidity, patient’s own wishes, the surgeon’s core medical values, expected natural course of non-operative treatment and priority considerations regarding the use of limited resources. Do objective clinical findings, such as radiology and laboratory results, have greater impact on decision-making than “soft” clinical variables? In this study we investigate the parameters that surgeons consider significant in decision-making in cases of suspected appendicitis; specifically we describe the process leading to surgical intervention in real settings.

The purpose of the study was to explore the process behind the decision to undertake surgery on a patient with suspected appendicitis as a model for decision-making in surgery.

**Methods:**

All appendectomy procedures (*n* = 201) at the Department of Surgery at Karolinska University Hospital performed in 2009 were retrospectively evaluated. Every two consecutive patients seeking for abdominal pain after each case undergoing surgery were included as controls. Signs and symptoms documented in the medical records were registered according to a standardized protocol. The outcome of this retrospective review formed the basis of a prospective registration of patients undergoing appendectomy. During a three- month period in 2011, the surgeons who made the decision to perform acute appendectomy on 117 consecutive appendectomized patients at the Karolinska University Hospital, Huddinge, and Södersjukhuset, were asked to answer a questionnaire about symptoms, signs and diagnostic measures considered in their treatment decision. They were also asked which three symptoms, signs and diagnostic measures had the greatest impact on their decision to perform appendectomy.

**Results:**

In the retrospective review, tenderness in the right fossa had the greatest impact (OR 76) on treatment decision. In the prospective registration, the most frequent symptom present at treatment decision was pain in the right fossa (94 %). Tenderness in the right fossa (69 %) was also most important for the decision to perform surgery. Apart from local status, image diagnostics and blood sample results had the greatest impact.

**Conclusion:**

Local tenderness in the right fossa, lab results and the results of radiological investigations had the greatest impact on treatment decision.

## Background

Effective health care requires rapid and effective decision-making. In ideal circumstances this implies careful consideration of key factors before the decision is made. Important factors to consider, for example, are the safety of diagnostic and treatment alternatives as well as the impact of the decisions taken on patient safety, quality-of-life, health economics and, in some cases, long-term survival. It is difficult, however, to base each individual decision on a complete analysis of all relevant factors, and in the emergency department many decisions are probably made based on the outcome of previous similar cases - “pattern recognition”.

Acute appendicitis is one of the most common acute abdominal conditions. The decision to perform surgical exploration in suspected appendicitis involves diagnostic accuracy, patient age and co-morbidity, patient’s own wishes, the surgeon’s core medical values, expected natural course of non-operative treatment and priority considerations regarding the use of limited resources. The decision to operate a patient with suspected appendicitis can therefore serve as a model to study how various clinical factors are ranked in surgical decision-making.

Under optimal circumstances, surgeons aim for high sensitivity, with as few neglected appendicitis diagnoses as possible. At the same time one hopes for high specificity, with as few negative explorations as possible. It has recently been questioned if it’s enough just to make the right diagnosis, or if it’s also necessary to evaluate whether it’s a gangrenous appendicitis that may heal itself or if it’s a progressive appendicitis with a high risk for perforation. The surgeon is thus expected not only to make a correct diagnosis but also to stage the condition based on rather limited facts. How do surgeons think when making these decisions? Do objective clinical findings, such as radiology and laboratory results, have greater impact on decision-making than “soft” clinical variables? Is there adherence to both experience and to evidence-based surgery?

There are several examples of studies [1, 2] comparing different means of diagnosis in appendicitis, but few have identified the intellectual process involved in the decision to perform appendectomy. The purpose of this study was to investigate parameters that surgeons consider significant in decision-making in cases of suspected appendicitis.

## Methods

The study was performed in two stages. At first, a retrospective study aimed at identifying the most important factors influencing the decision to perform appendectomy was performed. This was followed by a prospective study aimed at exploring the relative impact of each of these factors. The retrospective study was undertaken in order to identify relevant questions put to the surgeons in the prospective study, i.e., to create a questionnaire that was appropriate for the surgeons performing appendectomies in the chosen hospital setting. The decision process for all appendectomy procedures (*n* = 201) at the Department of Surgery at the Karolinska University Hospital, in 2009, was retrospectively evaluated. After each case operated upon, the next two consecutive patients that were seeking help for abdominal pain at the Emergency Department at the Karolinska University Hospital, served as controls. The evaluation focused on the signs and symptoms documented in the medical records at the time of admission and the time of surgery – all signs and symptoms in the patient charts were taken into account.

The prospective part was carried out over a 3-month period in 2011 at the Karolinska University Hospital, Huddinge, and at Södersjukhuset in Stockholm, two hospitals with emergency departments, taking care of more than 100 000 patients per year. Surgeons who made the decision to perform acute appendectomy were asked to fill in the questionnaire within 24 h after the appendectomy. The questionnaire included questions on symptoms, signs and diagnostic measures at the time of the decision to perform surgery. They were also requested to record which of the symptoms, signs and diagnostic measures that had the greatest impact on the decision to perform appendectomy (three positive findings per patient). The set of three symptoms or signs, was based on the first part of the study where the surgeons were requested to state which factors had the greatest impact on the decision to perform surgery.

The study does not involve any patient related intervention and it wasn’t reviewed by any Ethics Review Board.

### Statistics

Odds ratios for deciding on appendectomy in the first part of the study were estimated by dividing the odds for undergoing surgery in patients with affirmed sign, symptoms or diagnostic measure with odds for the patients for whom it was not affirmed. In the second part of the study, the prevalence of each affirmed sign, symptom and diagnostic measure was determined as well as the frequency of each of these that were allotted greatest impact on the decision to perform surgery.

## Results

Among the 201 patients in the retrospective study, there were 15 signs and symptoms that significantly divided patients with acute appendicitis from those without (Table 1). Tenderness in the right fossa was found to have the greatest impact on the decision to perform appendectomy with an odds ratio (OR) of 76, followed by raised CRP (OR 47), pain in the right fossa (OR 29), increasing CRP (OR 23), indirect tenderness (OR 19), pain migration (OR 18) and image diagnostics (OR 4) i.e., these items differed the most between patients with appendicitis and those without. These data were used to construct a new questionnaire in which the surgeon could choose between 25 items comprising all 15 with a significant odds ratio plus a few items that are commonly described in the literature on the diagnosis of acute appendicitis [3, 4].

**Table 1:** Outcome of the retrospective investigation

Symptoms	Odds ratio	95 % confidence interval	*p*
Nausea	2.64	1.73-4.03	<0.001
Vomiting	2.26	1.42-3.58	0.001
Loss of appetite	3.40	1.97-5.88	<0.001
Pain in the right fossa	23.03	13.25-40.05	<0.001
Pain in the left fossa	0.77	0.39-1.51	0.45
Pain in the right hypochondrium	0.27	0.12-0.62	0.002
Pain in the left hypochondrium	0.39	0.11-1.36	0.14
Pain in the epigastrium	0.73	0.45-1.19	0.21
Pain in the umbilical area	2.13	1.35-3.36	0.001
Pain migration	23.65	11.86-47.15	<0.001
Insidious occurrence of pain	1.31	0.79-2.17	0.30
Pain provoked by movement	2.04	1.12-3.72	0.019
**Signs**			
Tenderness in the right fossa	80.35	35.38-182.50	<0.001
Tenderness in the left fossa	0.95	0.47-1-90	0.88
Tenderness in the right hypochondrium	0.18	0.07-0.45	<0.001
Tenderness in the left hypochondrium	0.53	0.14-1.93	0.34
Tenderness in the epigastrium	0.16	0.07-0.36	<0.001
Tenderness in the umbilical area	0.87	0.47-1.61	0.66
Indirect tenderness	29.12	11.24-75.43	<0.001
Rigid abdomen	2.06	0.71-6.00	0.18
**Diagnostic measures**			
Image diagnostics	4.99	3.12-7.97	<0.001
Raised leukocyte count	11.11	6.55-18.84	<0.001
Raised CRP	28.86	15.19-58.82	<0.001
Increasing CRP	27.97	10.78-72.56	<0.001

Association between recorded symptoms, signs and diagnostic measures and the risk for subsequently undergoing appendectomy

Altogether 117 patients were included in the prospective study (66 women, 51 men). Mean age was 37 ± 16 years (± SD). The patients were operated at the Departement of Suregery at Södersjukhuset and at Karolinska University Hospital. The outcome of the prospective investigation regarding symptoms, signs and diagnostic measures recorded prior to surgery is presented in Table 2.

**Table 2:** Outcome of the prospective investigation

	Symptoms, signs and diagnostic measures present at time of treatment decision, per cent of all cases		Symptoms, signs and diagnostic measures with greatest impact on treatment decision (3 per patient), per cent of those with positive symptom, sign or diagnostic measure	
**Symptoms**	*n*	%	*n*	%
Nausea	66	56	0	0
Vomiting	39	33	2	5
Loss of appetite	40	34	4	10
Pain in the right fossa	110	94	27	25
Pain in the left fossa	13	11	0	0
Pain in the right hypocondrium	12	10	0	0
Pain in the left hypochondrium	2	2	0	0
Pain in the epigastrium	12	10	0	0
Pain in the umbilical area	29	25	4	14
Pain migration	66	56	33	50
Insidious occurrence of pain	58	50	4	7
Pain provoked by movement	55	47	10	19
**Signs**				
Fever	38	32	8	21
Tenderness in the right fossa	106	91	81	76
Tenderness in the left fossa	18	15	2	11
Tenderness in the right hypochondrium	7	6	1	14
Tenderness in the left hypochondrium	2	2	0	0
Tenderness in the epigastrium	2	2	0	0
Tenderness in the umbilical area	13	11	0	0
Indirect tenderness	51	44	18	35
Rigid abdomen	8	7	3	38
**Diagnostic measures**				
Image diagnostics	78	67	70	90
Raised leukocyte count	76	65	29	38
Raised CRP	89	76	31	35
Increasing CRP	41	35	13	32

Symptoms, signs and diagnostic measures recorded prior to surgery

In the prospective study the most frequent symptoms present at the time of decision to operate were pain in the right fossa (94 %), pain migration (56 %) and vomiting (56 %). The most frequent signs were tenderness in the right fossa (91 %), raised CRP (76 %) and image diagnostics (67 %).

Signs, symptoms, and diagnostic measures with greatest impact on treatment decision, according to the surgeon performing the appendectomy and recorded after the outcome of surgery was known (3 per patient), were tenderness in the right fossa (76 %), image diagnostics (90 %) and pain migration (50 %).

## Discussion

Acute abdominal pain is the cardinal symptom behind a vast number of abdominal conditions including several that require immediate surgical treatment. The ambition of the surgeon responsible is therefore to decide, as soon as possible, whether the underlying condition requires urgent or sub-acute surgical intervention. However, harmless or non-urgent problems may lie behind the same cardinal symptom. By employing cost- effective diagnostic measures avoiding unnecessary exposure of the patient to radiation, the challenge remains to identify those patients requiring emergency surgery from those who suffer from a less serious condition that may be treated conservatively and without time limitation. Dealing with such a highly complex decision-making process calls for a logically coordinated and systematic overall process [5]. It is understood that the diagnosis of appendicitis is based on a balanced evaluation of signs, symptoms and tests, though, how one arrives at this balanced judgement can be discussed. We have tried to see this from the individual surgeon’s point of view, i.e., we have analysed not how decision-making *should* be done, but rather *how* it is done.

The emergency department is a unique clinical milieu of inconstancy, uncertainty, variety, and complexity. In the emergency room setting the management of trauma and illness has a limited time perspective and is often carried out under pressure. This situation forces physicians to adopt a distinctive way of thinking [6, 7]. The aim of this study was to describe how decisions are taken under these circumstances.

When analyzing the various signs and symptoms of appendicitis we found that nausea and vomiting to some extent, were present in many cases, but had no impact on decision-making. The same was seen regarding loss of appetite. These symptoms were obviously thought of as being a sign that the patient was “ill” but not that the patient had an “appendicitis requiring surgery”. The only pain characteristics taken into account by the surgeon when deciding to operate were pain in the right lower fossa and pain migration (highest scored). Pain in the right fossa and indirect tenderness were the only signs that caused the surgeon to think of surgery, and rigidity of the abdominal wall – indicating a more severe peritonitis, but only found in 7 % of cases – was obviously overshadowed by pain in the right fossa (impact on decision to operate in 38 % versus 76 % resp.). Imaging studies (ultrasonography, computed tomography), however, had the greatest impact on the surgeons’ decision to operate. Even though these were performed in only two thirds of cases, results were an important basis for the decision to operate in most. In this study the findings of imaging studies were not registered, but we speculate that in most of the 70 cases the radiologic verdict was “appendicitis”. This would confirm that it is mentally difficult not to operate on a radiologically or ultrasonographically demonstrated sick appendix. This presents a problem since imaging does not always describe the truth and furthermore not all confirmed cases of appendicitis require surgery. It is also interesting to note that a raised white cell count and a raised and increasing CRP– were chosen by the surgeons as factors with high impact in only a third of cases when only the option was to choose three. This is interesting since all three are well-known to correlate with the degree of inflammation and therefore likelihood of acute appendicitis.

All factors included in the study were, to varying extent, factors that strengthened the decision to perform appendicitis. However, we did not include factors that decreased the probability of appendicitis. Such factors, e.g., gastrointestinal bleeding, gynecological symptoms and decreasing CRP and leukocytes, may also have a great impact on the decision process, although in the negative direction.

This study did not aim to define those factors of most importance for decision-making in cases of suspected appendicitis, but to gain insight into what makes the surgeon decide to operate. It must also be understood the surgeons in this study were not experienced experts with a certain interest in appendicitis and appendectomy. They were, in all but a few cases, surgeons under training (½ to 5 years prior surgery) working alone but with the back-up of a resident if needed (decision of the intern) not present at the Emergency Department, usually during on-call hours. This study thus describes reality in a Scandinavian surgical department, and not an ideal situation with highly experienced surgeons. Despite the presence of senior colleague, residents adhere to a hierarchy when seeking advice in clinical matters [8]. Furthermore, the cognitive processes employed by residents experienced in critical care are quantitatively and qualitatively different from those used by their junior counterparts; this is why the setting of our study is of importance [9].

The role of computed tomography and ultrasonography in cases of suspected appendicitis has recently been the subject of intensive discussion [10, 11]*.* To diagnose appendicitis, CT has a greater sensitivity and negative predictive value in older than in younger patients. CT is also associated with less negative appendectomy- rates for all female patients regardless age [12]. In this study two thirds of the patients underwent diagnostic radiography when appendicitis was suspected, but almost all surgeons considered that this was one of the three most important factors in decision-making. Nowadays imaging is performed in more than half of patients with suspected appendicitis, but despite this, surgeons continue to rank signs, symptoms, and laboratory results as the key factors leading to appendectomy. In this study, however, we cannot say if the radiological results (positive, negative, or equivocal) influenced the process of decision-making.

The decision to rely on image diagnostics as well as performing surgery based on data available at the first examination is, to a great extent, dependent on the age of the patient. With increasing age, the prevalence of pathological conditions (e.g., diverticulitis and colon tumours) mimicking appendicitis increases. This may have had an impact on the impact of image diagnostics on the decision to perform surgery.

The surgeons were requested to state what had the greatest impact on the treatment to perform surgery when the procedure was already completed. There is, however, a natural course in acute appendicitis. All exams are not always performed at the same time as blood samples are taken or CT scan. This may have had an impact on the registration, since diagnostic measures late in the course, when the diagnosis had become more obvious, may have been attributed a greater impact than those registered immediately after admission.

An interesting conclusion from this study is that the differences in frequency of symptoms, rather than the three symptoms without ranking were considered most important by the surgeon making the decision to take the patient to the surgery. Insidious occurrence of pain (50 %), pain provoked by movement (47 %) and elevated leukocyte count (76 %) are also high on the list of symptoms not being assigned an important predictive value by the surgeon. This is probably because these symptoms only indicate abdominal disease and are thus not specific for acute appendicitis.

It is also interesting to note that in only 25 % of the 110 cases with a history of pain in the right lower quadrant was included among the three most important signs and symptoms in these cases. On the other hand, 66 % (only!) of the 106 patients with pain on palpation in the right lower quadrant were considered to have appendicitis, i.e., this was among the three signs and symptoms the surgeons ranked highest. It is possible that the surgeon considered the results of his/her investigation more important than the patient history. It may also be that when choosing from the list of alternatives, patient history gave similar information but was omitted since the surgeon put more trust in his/her own clinical investigation.

It is also noteworthy that only half of the patients (*n* = 66) noted pain migration (from the umbilical areal to right lower fossa), but even more astonishing is that in only half of these cases the surgeon ranked that among the three most important signs and symptoms. In textbooks this is often portrayed as being a pathognomonic sign of appendicitis. The insidious occurrence of pain and pain provoked by movement also made little impression on the surgeons.

Fever and indirect tenderness were fairly uncommon signs among our patients, and were also given low diagnostic value by the attending surgeon. Moreover raised leukocyte count, raised CRP, and increasing CRP were only seen in 76, 89, and 41 % of patients respectively. In these cases these three signs were ranked by the surgeon to be amongst the three most valuable signs in only 38, 34, and 32 % of cases. Maybe it would have been more suitable to ask for normal CRP and leukocyte counts, that usually may exclude appendicitis, (if not measured too early) in the cause of the disease.

The present study reveals that decision-making in patients with appendicitis is largely based on “hard” data such as lab results and the results of radiographic investigations. It seems that bedside clinical skill has come under pressure, be it right or wrong. There is evidence that computed tomography, for example, has a higher accuracy [13] than the best clinical scores for diagnosing acute appendicitis [14]. Perhaps our dependence on signs and symptoms – once the gold standard – should be re-evaluated. There are many publications that have scrutinized the various aspects of initial assessment and emergency management of acute abdominal pain. The large body of evidence, however, seems to miss articles that describe a formally correct priority- and problem-based approach [15]. Considerable evidence suggests that wide regional variation exists in the service received by patients. Evidence-based guidelines that incorporate quality-of-life and patient preference may help address this problem. Systematic cost-effectiveness analyses may be used to improve resource allocation decisions [16]. However, clinical decision-making has, until now, always been the cornerstone of high-quality care in emergency medicine. The intensity of decision- making in this unique milieu is unusually high, and a combination of strategies has, of necessity, evolved to cope with the load. Cognitive short-cutting strategies may be especially adaptive in situations with time and resource limitations that prevail in many emergency departments, but occasionally these fail. Detection and recognition of these cognitive phenomena must be a first step in achieving cognitive de-biasing to improve clinical decision-making in the Emergency Department [17].

The study has some important limitations. The most important is that patients not operated upon are excluded. When doing the next study the unoperated, should be studied as well. The other factor that should be handled differently is the result of the imaging; the way a positive finding influence, if the patient should be or not, might be quite different from a negative one.

The present study was not aimed at providing any definite confirmation regarding the correctness of the decision. The endpoint was the decision to perform surgery, not the outcome of the procedure. Accordingly, we have not considered the final outcome in terms of clinical parameters or histopathologic examination, Although this information may have served as a corroboration of the clinical decision, the purpose of the study was to study the decision process, not what it finally lead to.

## Conclusion

In conclusion, we have found that decision-making in cases of suspected appendicitis is largely based on a systematic approach where “hard” data such as lab results and the results of radiological investigations play a major role and the assessment of signs and symptoms (“bedside clinical skill”) has less impact on decision-making, than is usually described in textbooks. The surgeon’s own feeling, intuition and experience must also be taken into account. How these various approaches are best merged remains unanswered, and if not dealt with in the near future there is a risk that the importance of bedside clinical skill will vanish in favour of “hard” data.
